# Morphometric characterization and temporal temperature measurements during hepatic microwave ablation in swine

**DOI:** 10.1371/journal.pone.0289674

**Published:** 2023-08-04

**Authors:** Nicole A. Varble, Ivane Bakhutashvili, Sheridan L. Reed, Jose Delgado, Zoi Tokoutsi, Bruno Frackowiak, Marco Baragona, John W. Karanian, Bradford J. Wood, William F. Pritchard

**Affiliations:** 1 Center for Interventional Oncology, Radiology and Imaging Sciences, Clinical Center, National, Institutes of Health, Bethesda, Maryland, United States of America; 2 Philips, Best, The Netherlands; 3 Fischell Department of Bioengineering, University of Maryland, College Park, Maryland, United States of America; 4 Bioengineering and National Cancer Institute Center, Bethesda, Maryland, United States of America; Fondazione Policlinico Universitario Agostino Gemelli IRCCS, ITALY

## Abstract

**Purpose:**

Heat-induced destruction of cancer cells via microwave ablation (MWA) is emerging as a viable treatment of primary and metastatic liver cancer. Prediction of the impacted zone where cell death occurs, especially in the presence of vasculature, is challenging but may be achieved via biophysical modeling. To advance and characterize thermal MWA for focal cancer treatment, an *in vivo* method and experimental dataset were created for assessment of biophysical models designed to dynamically predict ablation zone parameters, given the delivery device, power, location, and proximity to vessels.

**Materials and methods:**

MWA zone size, shape, and temperature were characterized and monitored in the absence of perfusion in *ex vivo* liver and a tissue-mimicking thermochromic phantom (TMTCP) at two power settings. Temperature was monitored over time using implanted thermocouples with their locations defined by CT. TMTCPs were used to identify the location of the ablation zone relative to the probe. In 6 swine, contrast-enhanced CTs were additionally acquired to visualize vasculature and absence of perfusion along with corresponding post-mortem gross pathology.

**Results:**

Bench studies demonstrated average ablation zone sizes of 4.13±1.56cm^2^ and 8.51±3.92cm^2^, solidity of 0.96±0.06 and 0.99±0.01, ablations centered 3.75cm and 3.5cm proximal to the probe tip, and temperatures of 50 ºC at 14.5±13.4s and 2.5±2.1s for 40W and 90W ablations, respectively. *In vivo* imaging showed average volumes of 9.8±4.8cm^3^ and 33.2±28.4cm^3^ and 3D solidity of 0.87±0.02 and 0.75±0.15, and gross pathology showed a hemorrhagic halo area of 3.1±1.2cm^2^ and 9.1±3.0cm^2^ for 40W and 90W ablations, respectfully. Temperatures reached 50ºC at 19.5±9.2s and 13.0±8.3s for 40W and 90W ablations, respectively.

**Conclusion:**

MWA results are challenging to predict and are more variable than manufacturer-provided and bench predictions due to vascular stasis, heat-induced tissue changes, and probe operating conditions. Accurate prediction of MWA zones and temperature *in vivo* requires comprehensive thermal validation sets.

## Introduction

Focal or regional thermal ablation, where application of heat may be used to induce cell death, is emerging as a viable treatment of primary and metastatic liver cancer [1, 2]. Specifically, microwave ablation (MWA) is becoming an alternative to surgical resection as a primary treatment or in conjunction with radiation, chemotherapy, or surgery [3–5]. Compared to alternatives such as radiofrequency ablation, MWA has superior energy and heat propagation and can deliver higher and more uniform heating in liver tissue [6, 7]. However, predicting the zone where cell death occurs is challenging, especially in the presence of the heat sink effect of tissue perfusion and blood flow in nearby vasculature.

Effective delivery of energy-based thermal ablation via microwaves requires adequate coverage of the lesion via a planned treatment volume and avoidance of critical structures. Ablation planning software and needle guidance technology may help ensure precise placement of probes, but subsequent MWA effects remain somewhat unpredictable. Previous studies have proposed workflows for optimization of probe trajectory to maximize tumor coverage or avoid critical structures [8–10]. However, accurate prediction of the subsequent ablation zone require consideration of complex interactions such as blood flow, tissue perfusion, and changes in tissue properties during ablation. Although MWA is less susceptible than radiofrequency ablation, any thermal modality is subject to perfusion-mediated vascular cooling, or the convective “heat-sink effect” across different scale vasculature [11], which can alter the anticipated ablation volume [12] and lead to miscalculation of treatment effectiveness [13, 14]. Minor changes to probe position, power, and ablation time are challenging to cognitively infer and could have major treatment implications, thus, comprehensive planning software may add value in patient treatment [15, 16].

Prior studies have used one or a limited combination of monitoring techniques to improve understanding of thermal ablation. These studies include thermal monitoring in phantoms or in perfused liver models [17, 18], computed tomography (CT), ultrasound, magnetic resonance imaging [19], or histopathologic analysis on ex vivo and in vivo swine models [20, 21]. To validate computational models of MWA, other studies have used a combination of approaches to examine focused hypotheses such as the heat-sink effect [22], internal water vaporization [23], tissue contraction [24], and the impact of simultaneous versus sequential power delivery [25]. However, validation of complex patient-specific planning for MWA may require comprehensive in vivo data with thermal monitoring, vascular and ablation zone imaging, and corresponding pathology in bench and in vivo models to better inform, validate, and predict ablation outcomes. Therefore, the objective of this study was to develop a comprehensive study method and collect, evaluate, and characterize MWA for the assessment of biophysical models designed to dynamically predict the ablation zone parameters, given the delivery device, power, probe location, and proximity to vessels.

## Materials and methods

### Study design and model overview

MWA temperature was monitored using thermocouples implanted in standardized locations with CT used to define their location relative to the ablation probe. A tissue-mimicking thermochromic phantom (TMTCP) which undergoes a temperature-dependent color change was used to identify the expected location of the ablation zone relative to the probe tip. The ablation zone size, shape, and temperature were characterized and monitored in the absence of perfusion in *ex vivo* liver. In swine, additionally, contrast-enhanced CTs were acquired to visualize vasculature and the devascularized ablation zone, along with corresponding post-mortem gross pathology.

### Microwave ablation and thermal measurements

Microwave ablation was performed for 10 minutes at 40 or 90 watts (W) with a single, clinically available, probe and system (Certus^LK^ ablation probe, 15cm length, 17 gauge, with Certus 140^™^ 2.45 GHz system, NeuWave, Johnson and Johnson, New Brunswick, NJ). Power settings were based on common clinical use, the manufacturer’s instructions and expected lesion size differences. Thermocouples (IT-18, Braintree Scientific, Inc. Braintree, MA) were calibrated and confirmed accurate ±0.2°C. Up to 8 thermocouples (denoted TC1 to TC8) were advanced to various pre-planned depths using a 17.6cm 17-gauge coaxial introducer needle and a custom 3D-printed template grid with conduits at 4mm increments. Temperatures were recorded at 1-second intervals (LogMaster OctTemp Logger, ThermoWorks, American Fork, UT) before, during and following MWA.

### Bench studies

TMTCP [26, 27], and bovine liver were studied within a 500mL container placed in an 18L water bath maintained at 37°C. Liver was placed in a 10% PBS solution prepared with deionized water. The MWA probe was advanced to the center of the TMTCP phantom or *ex vivo* bovine liver to an approximate depth of 8cm. Thermocouples were placed near the MWA probe tip, near the manufacturer’s described emitting point and at the proximal end of the predicted ablation zone (approximately 2cm and 4cm proximal to the tip, respectively).

Following MWA, CT images (Brilliance MX8000 IDT 16-section detector CT; Philips, Andover, MA) were acquired to identify the location of the thermocouples and probe and were reconstructed as 1mm sections at 0.5mm intervals. CT was acquired with both the probe and thermocouples in place and then with the probe removed to visualize the remaining thermocouples to eliminate metal artifact.

The TMTCP and liver were sectioned along the axis of the probe. Images of the TMTCP ablation were taken under controlled lighting conditions and exposure in a portable photography lightbox (SANOTO Softbox MK40, Whittier, CA). Images were color-corrected, and temperature contours were estimated using a previously validated technique that correlates color change and peak temperature [27]. The color edge was expected at approximately 60°C and was identified as the edge of the ablation zone. For bovine liver, the ablation zone was identified as the region where tissue drastically changed color and apparent density. Ablation zones were assumed to be axisymmetric.

### Preclinical *in vivo* studies

**Fig 1:**
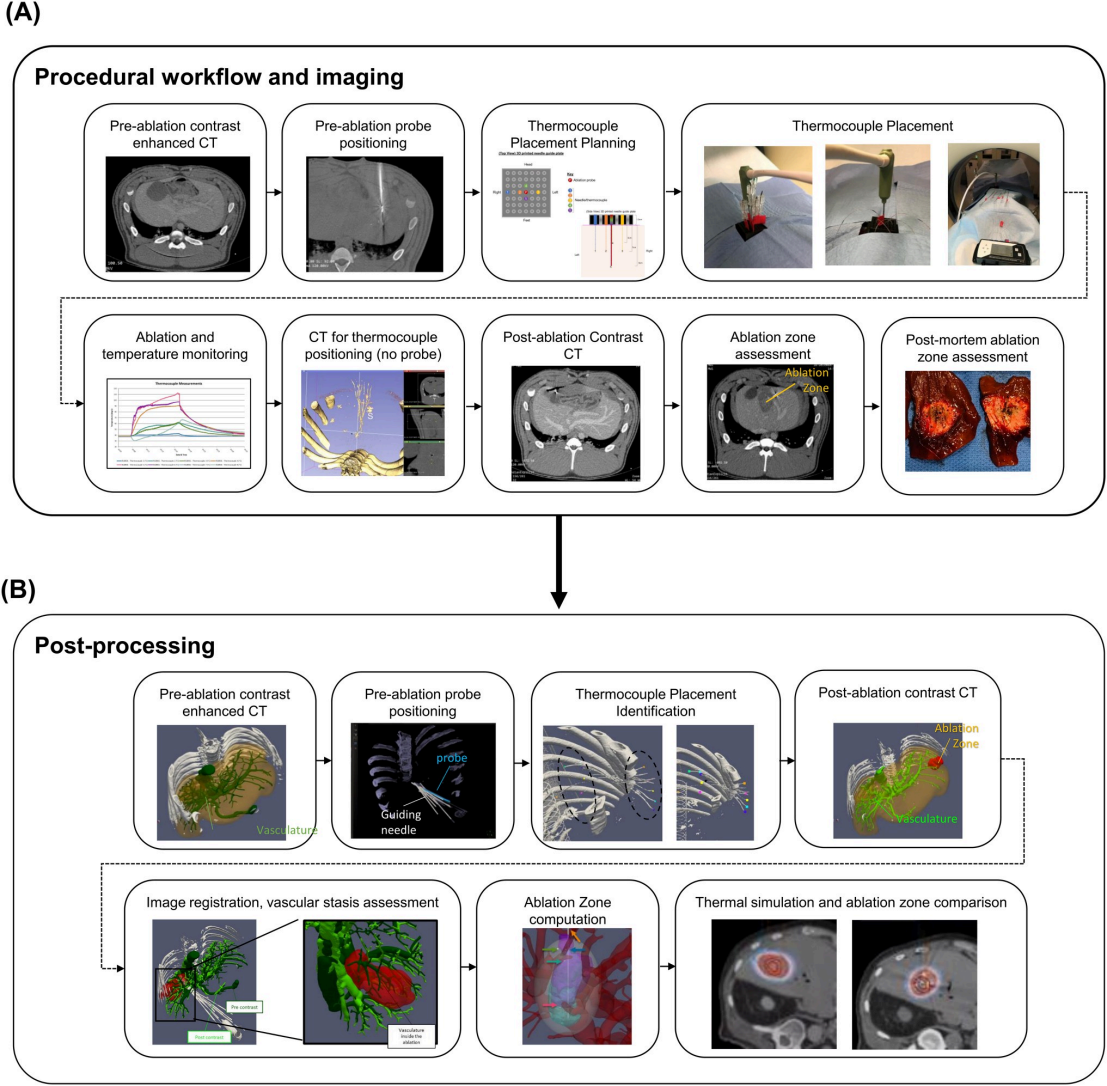
Procedural workflow, imaging, and post-processing steps towards validation of a patient-specific thermal model. (A) Procedural workflow. A pre-ablation contrast-enhanced CT was taken to plan for the ablation probe positioning. A general pattern for microwave ablation probe and thermocouple placement was planned and placed with assistance from a 3D printed grid. During ablation, temperatures were monitored. Immediately following ablation, a CT scan was taken both with and without the ablation probe in place, which could be used to co-localize thermocouples in relation to the ablation zone. After removal of the thermocouples, a post-ablation contrast-enhanced CT was taken to visualize both vessels and the ablation zone (non-perfusion zone). The liver was excised, and the ablation zone was identified and measured on cross-sectional slices that were cut perpendicularly to the axis of the probe. (B) During post-processing, the liver, vasculature, probe, and thermocouple locations can be identified. After image-registration, vasculature can be registered to identify vascular stasis. The segmented ablation zone and the probe location can then be used for comparison of expected and actual temperatures and ablation zones. A thermal simulation model can be run, based on the surrounding segmented tissue, and isotherms can be calculated and compared.

The *in vivo* procedural workflow is shown in Fig 1A. Five healthy swine (67±6.8kg; Oak Hill Genetics, Ewing, IL) were studied under a protocol approved by the National Institutes of Health Institutional Animal Care and Use Committee. The swine were sedated with intramuscular ketamine (25 mg/kg), midazolam (0.5 mg/kg), and glycopyrrolate (0.01 mg/kg) and anesthetized with propofol (1 mg/kg intravenously). The subjects were intubated, mechanically ventilated, and maintained under general anesthesia with isoflurane (1%–5%) (Isoflo; Abbott Animal Health, North Chicago, IL) and 100% oxygen throughout the procedure. The stomach was decompressed by needle gastrostomy, if needed. Breath holds at inspiration with pressure fixed at 20 cm H_2_0 was used for each CT scan as well as during insertion of the MWA probe and the needles for thermocouple placement. Three to 6 1.5 mm metal beads (McMaster-Carr, Los Angeles, CA) were inserted in the liver via a biopsy introducer set under ultrasound guidance to serve as registration fiducials. The beads were placed in different axial planes and at various depths away from the planned ablation zone so they would not contribute to tissue ablation effects.

Multiphase contrast-enhanced CT imaging with bolus tracking was performed with 5, 30, and 50 second delays for the arterial, portal and venous phases, respectively, during power injection (MEDRAD Stellant CT Injection System; Bayer Healthcare, Leverkusen, Germany) of 80 mL of iopamidol (ISOVUE-370, Bracco Diagnostics, Monroe Township, NJ,) at 4 mL/s followed by saline, 30 mL at 4 mL/s. MWA probe and thermocouple placement was planned based on the hepatic anatomy including vasculature and identification of an access window to permit insertion using the template grid. The goal was placement such that major vessels would not be contained within the expected ablation zone, typically in the central liver with transabdominal access at an average depth of 10.6±1.2cm (range 9-12cm). A 4x4 cm area of the skin was resected and a 3.2x3.2 cm 3-D printed grid was positioned on the subcutaneous tissue and sutured in place to guide probe and thermocouple insertion. After insertion of the probe, the coaxial introducer needles were inserted, the thermocouples were advanced through the needles to the tips, and then the needles were withdrawn over the thermocouple wires while maintaining the position of the wires. Multiple non-contrast CT scans were acquired during the stages of probe and thermocouple insertion.

MWA was performed at 40W and 90W: a single 40W (n = 2) or 90W ablation (n = 2) was performed in four subjects, and both ablations (n = 1 each) were performed in one subject. Four subjects had one ablation due to liver size and study timeline constraints. Temperature measurement began approximately 3 minutes before MWA and continued for 5 minutes after MWA completion.

Following MWA, non-contrast CT scans were acquired before removal of the MWA probe and after probe removal with the thermocouples in place. Thermocouples were removed and a final multiphasic contrast-enhanced CT was acquired to visualize the vasculature and define the estimated immediate ablation zone by volume segmentation, based on image intensity related to perfusion. In one case, post-ablation images were acquired with cone-beam CT (Allura Xper FD20; Philips) using roll CBCT protocols (120 kV, 60 frames per second for 8 seconds, 480 images, +90 to -90 degree) and the same contrast injection scheme. Euthanasia was performed approximately 2.5 hours after MWA to ensure standardization between subjects by intravenous administration of a combination of pentobarbital sodium 390 mg/ml and phenytoin sodium50 mg/mL (Beuthanasia-D).

### Data analysis

#### Image registration and probe and thermocouple locations

CT scans were co-registered using a rigid, mutual information algorithm in ITK-SNAP (v3.6.0, http://www.itksnap.org/) for bench studies, or with fast elastic image registration for *in vivo* studies (IntelliSpace Portal, version 10.1, Philips). The cylindrical coordinates of the thermocouples were computed as radial distance (r) from the probe shaft and the longitudinal distance (z) to the probe tip. This was accomplished by coordinate transformation, where the probe was aligned with the z-axis and the tip was defined as the origin. The MWA system provided internal probe temperature measurements from a thermocouple located 4cm from the ablation probe tip. Data obtained from probe and thermocouple locations were compared to the manufacturer-provided expected ablation profiles at 40W and 90W for 10 minutes, which was obtained from *ex vivo* bovine liver studies, along with the internal probe temperature. According to the manufacturer, the centroid of the manufacturer-provided ablation zone was 2cm proximal to the ablation probe tip and volume was estimated based on the volume of an ellipsoid. Based on size and emitting point, for 40W ablations, the manufacturer-provided ablation zone ends just proximal to the probe tip, and for the 90W ablations, the expected zone extends beyond the probe tip by 1.5cm.

#### 2D and 3D ablation zone morphometrics

For both bench and *in vivo* ablation zones the 2D cross-sectional areas were calculated and compared to the manufacturer-provided expected ablation zones. To describe the irregularity of the cross-sectional slices, the shape index, solidity, or the ratio of area to the convex area, were calculated in ImageJ (imagej.nih.gov/ij/).

For 3D analysis of the morphometrics of the ablation zone *in vivo*, the non-perfused zone was segmented in ITK-SNAP [28]. The segmented ablation zone was defined and estimated as the low attenuation, nonperfused region on the portal phase contrast CT. The volume and image intensities were recorded. 3D solidity was calculated as the ratio of the ablation volume to the convex hull volume. The convex hull of the ablation zone was computed in Blender (Stitching Blender Foundation, Amsterdam, The Netherlands: http://www.blender.org). The centroid of the segmented ablation volume was registered with the centroid of the manufacturer-provided ablation volume and compared. The differences between the two registered volumes were visualized using CloudCompare (cloudcompare.org). An unpaired t-test was used to compare morphometrics of 40W and 90W ablations.

#### Gross pathology

The ablation zone was identified in the excised liver by the investigators. Approximately 5mm thick sections were cut perpendicular to the axis of the probe. Three regions of the heat-affected zone were identified by characteristic concentric areas as defined by Meloni et al. including: (i) central coagulative necrosis, (ii) region of irreversibly damaged hepatocytes, and (iii) outer hemorrhagic halo, which was considered the edge of the ablation zone [21].

## Results

### Bench studies

#### Ablation zone location and size

**Fig 2:**
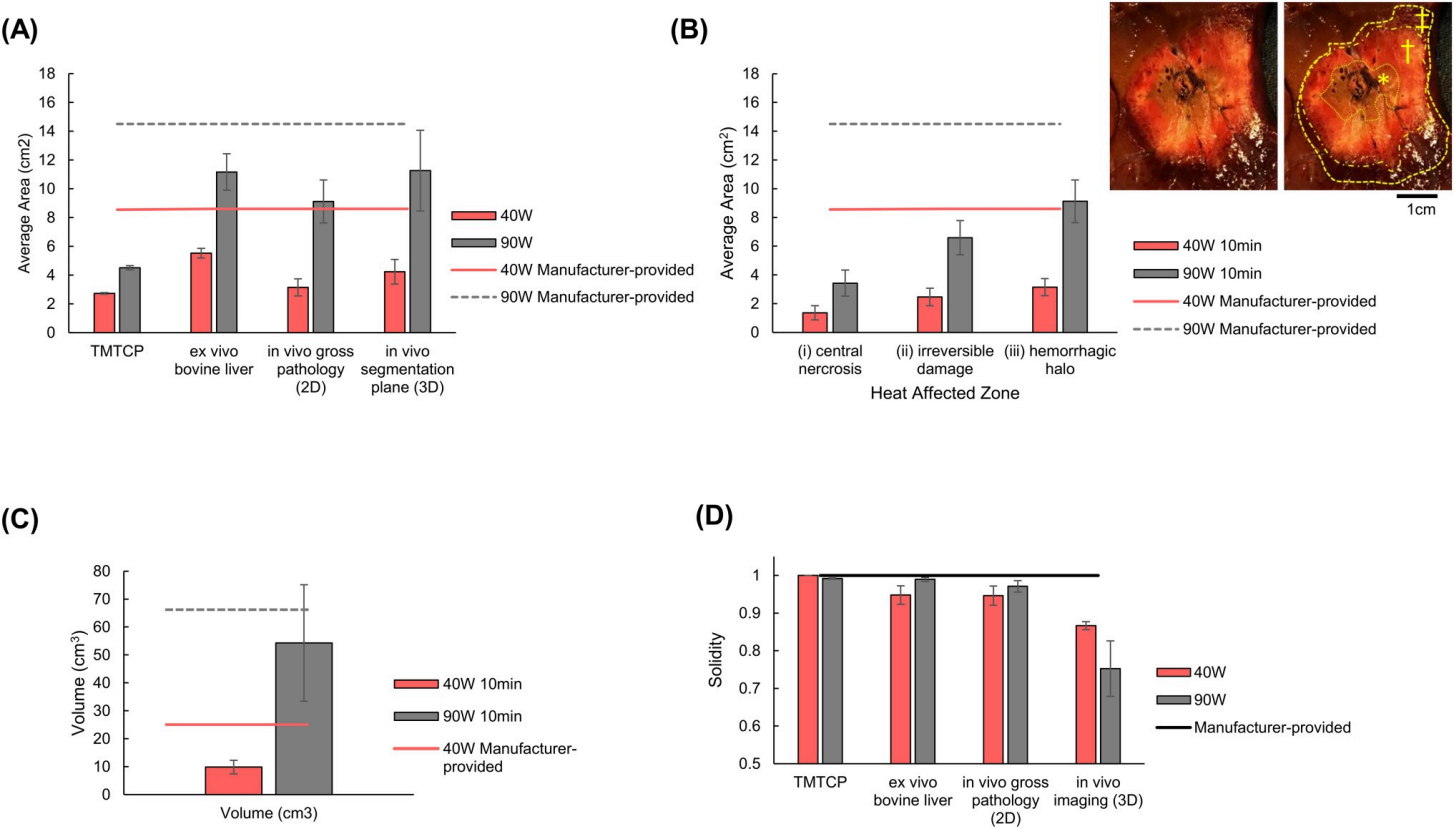
Morphometric results from *ex vivo* and *in vivo* studies for 40W (red) and 90W (gray) 10-minute microwave ablations with comparison to manufacturer-provided expected MWA volumes (red and grey lines). (A) Average largest cross-sectional area (section perpendicular to the probe track) for tissue mimicking thermochromic phantom (TMTCP), *ex vivo* bovine liver, and *in vivo* swine gross pathology. (B) Average cross-sectional area on gross pathology following *in vivo* ablation. The inset shows a representative cross-section including areas of central necrosis (*), irreversible coagulative damage (†) and hemorrhagic halo (‡), with the outer boundary of each zone outlined by a yellow hatched line. (C) Volume of the *in* vivo ablation defined as non-enhancement on post-ablation contrast CT. (D) Solidity of the ablation zone in cross-sectional planes for TMTCP and *ex vivo* bovine liver or the 3D *in vivo* images in swine.

**Fig 3:**
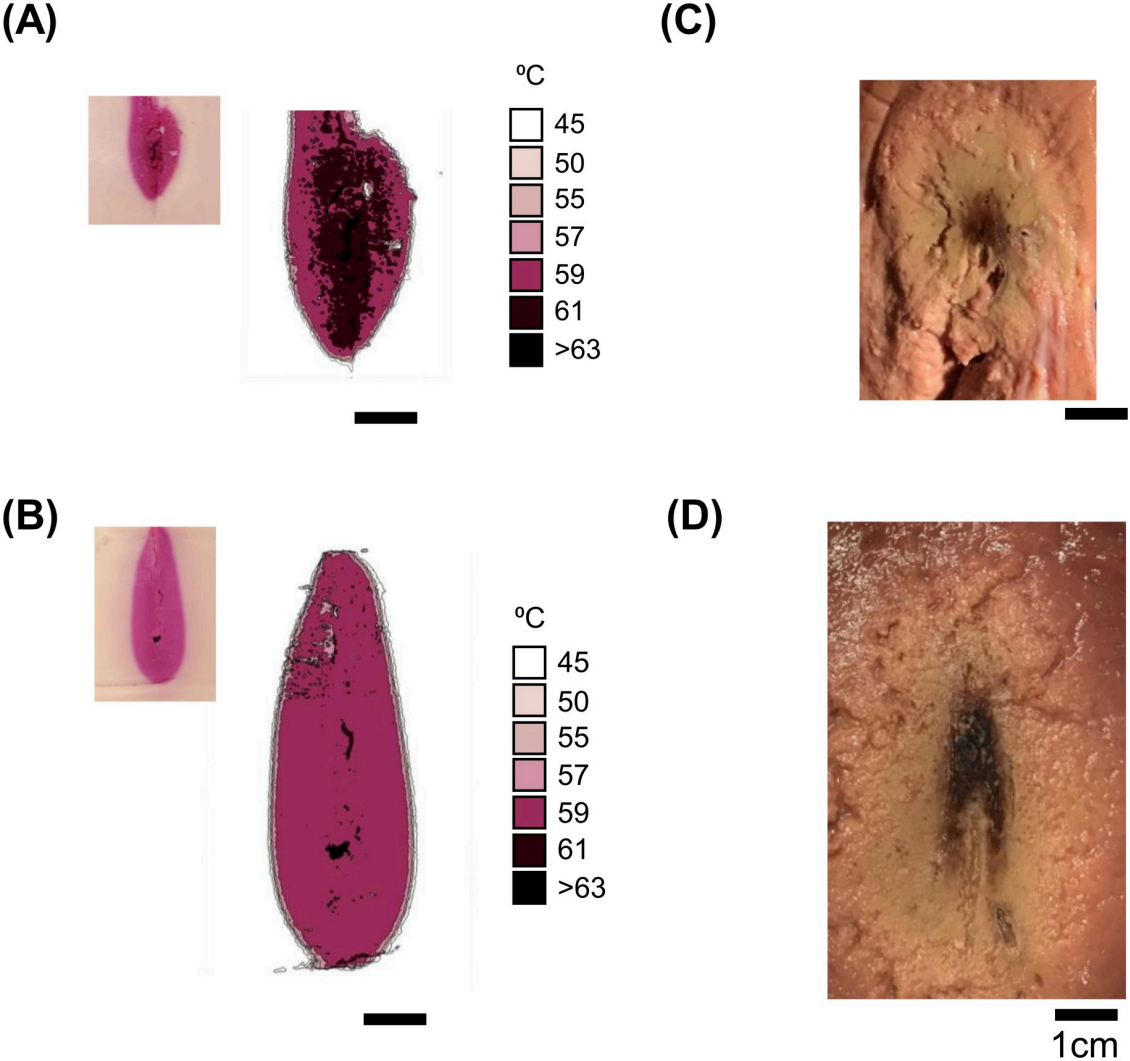
Longitudinal sections of thermal ablation in tissue-mimicking thermochromic phantom and *ex vivo* bovine liver following 10-minute 90W and 40W microwave ablation protocols. (A, B) Tissue-mimicking thermochromic phantom response for 40W and 90W ablations. A longitudinal cut along the axis of the probe track reveals quantified temperature contours. The inset show the unprocessed raw image. (C, D) *Ex vivo* bovine liver ablation with similar longitudinal cut along the axis of the probe track for 40W and 90W ablations. The microwave ablation probes were inserted from above.

The ablation zones were smaller than manufacturer-provided zones in longitudinal sections in both the TMTCP and *ex vivo* bovine liver (Fig 2A, Table 1). In the TMTCP, ablation areas were 2.73±0.11cm^2^ and 4.51±0.30cm^2^ compared to the manufacturer-provided ablation zone of 8.6cm^2^ and 14.5cm^2^ for 40W and 90W, respectively. The temperature gradients (Fig 3) were maximal closest to the ablation probe shaft with centroids 3.0cm and 3.4cm from the ablation probe tip and the distal end of the ablation zone was 0.75cm and 0cm proximal to the probe tip for the 40W and 90W ablations, respectively. In *ex vivo* bovine liver, ablation zone areas were 5.53±0.67cm^2^ and 11.17±2.53cm^2^ for the 40W and 90W ablations, respectively.

**Table 1:** Ablation size and morphometrics for 40W and 90W 10-minute ablations for bench and *in vivo* studies.

		Area (2D, cm^2^) or Volume (3D, cm^3^)	Solidity (-)
		*Average ± SD*	*Average ± SD*
**40W 10min**	TMTCP (2D)	2.7 *± 0*.*11*	1.00 *± 0*.*00*
	*ex vivo* bovine liver (2D)	5.5 *± 0*.*67*	0.95 *± 0*.*049*
	*in vivo* gross pathology (2D)	3.2 *± 1*.*2*	0.95 *± 0*.*051*
	Manufacturer-provided (2D)	8.6	1.0
	*in vivo* imaging (3D)	9.8 *± 4*.*8*	0.87 *± 0*.*021*
	manufacture-provided (3D)	24.5	1.0
**90W 10min**	TMTCP (2D)	4.5 *± 0*.*30*	0.99 *± 0*.*0035*
	*ex vivo* bovine liver (2D)	11 *± 2*.*5*	0.99 *± 0*.*011*
	*in vivo* gross pathology (2D)	9.1 *± 3*.*0*	0.97 *± 0*.*030*
	manufacturer-provided (2D)	14.5	1.0
	*in vivo* imaging (3D)	54 *±* 42	0.75 *± 0*.*15*
	manufacture-provided (3D)	60	1.0

SD = standard deviation, TMTCP = tissue mimicking thermochromic phantom

In the TMTCP model solidity was 1.00±0.00 and 0.99±0.01 for 40W and 90W ablations, respectively (Fig 2D, Table 1). In the *ex vivo* bovine liver, solidity was 0.95±0.05 and 0.99±0.01 for 40W and 90W ablations, respectively.

#### Temporal temperature measurements

**Fig 4:**
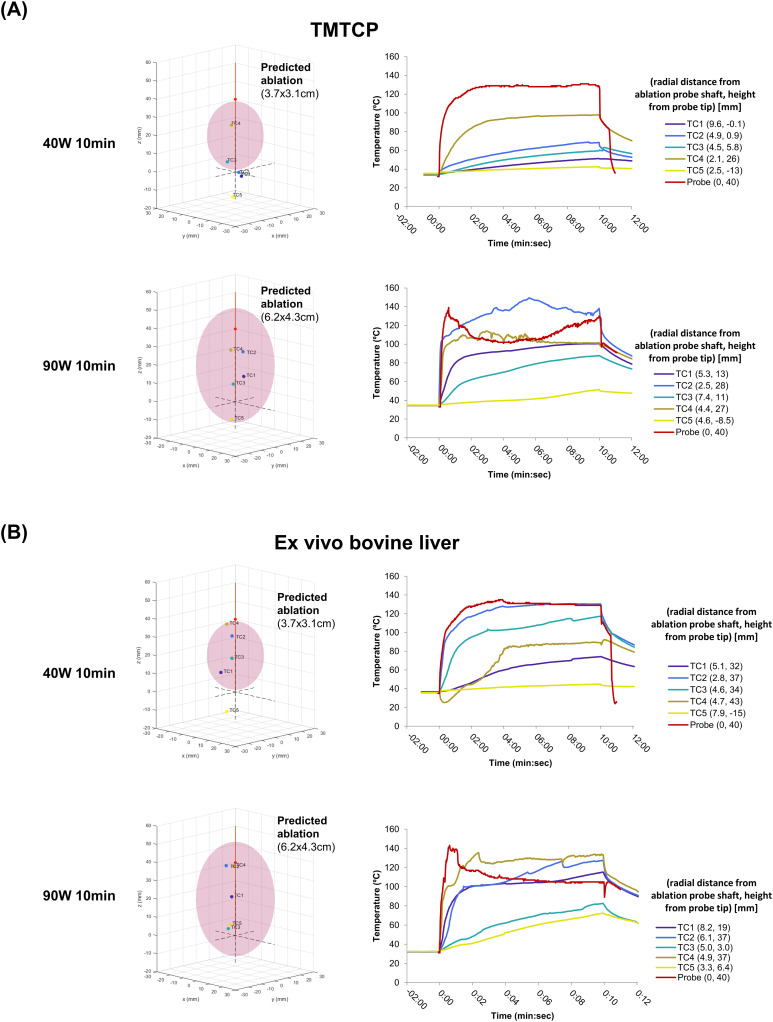
Probe location and temporal temperature measurements for 40W and 90W microwave ablation in TMTCP and *ex vivo* bovine liver. (A) TMTCP and (B) *ex vivo* liver with manufacturer-provided ablation zones (pink) shown on the right for 40W and 90W ablations. The position of the thermocouples relative to the ablation probe are indicated. Temporal temperature measurements (right) from thermocouples at varying distances from shaft and tip are shown, with color indicating thermocouple number. The variable radial distance from the ablation probe shaft (r) and height from the ablation probe tip (z) are in parentheses. The temperature internal to the probe itself (red) is provided by the system. TC = thermocouple.

The locations of the thermocouple tips were identified relative to the ablation probe and associated with the corresponding temperature curves collected during the ablation (Fig 4, Table 2). Maximum thermocouple temperatures were reached at the end of the 10-minute ablation period. Thermocouples that were most central to the ablation reached 50 ºC at 14.5±13.4s and 2.5±2.1s and 60ºC at 26.0±24.0s and 4.5±3.5s for 40W and 90W ablations, respectively. These thermocouples were located closest to the ablation probe shaft at 2.6 and 3.7cm proximal to the ablation probe tip for the 40W and 90W ablations, respectively.

**Table 2:** Thermocouple location and maximum temperature achieved during 40W and 90W 10-minute ablation protocols for tissue-mimicking thermochromic phantom and *ex vivo* bovine liver studies.

Bench test setting	Ablation protocol	Thermocouple	r (mm)	z (mm)	Maximum Temperature (°C)
**TMTCP**	40W 10min	1	9.6	-0.1	51
		2	4.9	0.9	69
		3	4.5	5.8	63
		4	2.1	26	98
		5	2.5	-13	46
		Internal to Probe	0	40	131
	90W 10min	1	5.3	13	101
		2	2.5	28	149
		3	7.4	11	88
		4	4.4	27	114
		5	4.6	-8.5	52
		Internal to Probe	0	40	139
**Ex vivo bovine liver**	40W 10min	1	5.1	32	74
		2	2.8	37	131
		3	4.6	34	117
		4	4.7	43	92
		5	7.9	-15	68
		Internal to Probe	0	40	131
	90W 10min	1	8.2	19	115
		2	6.1	37	128
		3	5	3	83
		4	4.9	37	136
		5	3.3	6.4	72
		Internal to Probe	0	40	143

The radial distance from the probe (r) and distance from the probe tip (z) were defined. The maximum temperatures were recorded closest to the ablation probe shaft (r~0) and at 2.6 and 3.7 cm from the ablation probe tip (z), for 40W and 90W ablations, respectively. The probe thermocouple is internal to the MWA system.

Three of four thermocouples placed outside of the manufacturer-provided 40W ablation zone in TMTCP and *ex vivo* bovine liver reached maximum temperatures between 51 and 68°C, although for <1 min. One thermocouple during the 90W TMTCP study was in the manufacturer-provided ablation zone, but only reached a maximum temperature of 52°C.

### Preclinical *in vivo* studies

For the in vivo studies, thermocouples that were most central to the ablation reached 50ºC at 19.5±9.2s and 13.0±8.3s and 60ºC at 41.0±17.7s and 23.0±17.1s for 40W and 90W ablations, respectively. Maximum temperatures occurred at the end of the ablation (10mins).

#### 3D segmentation of ablation zone

**Fig 5:**
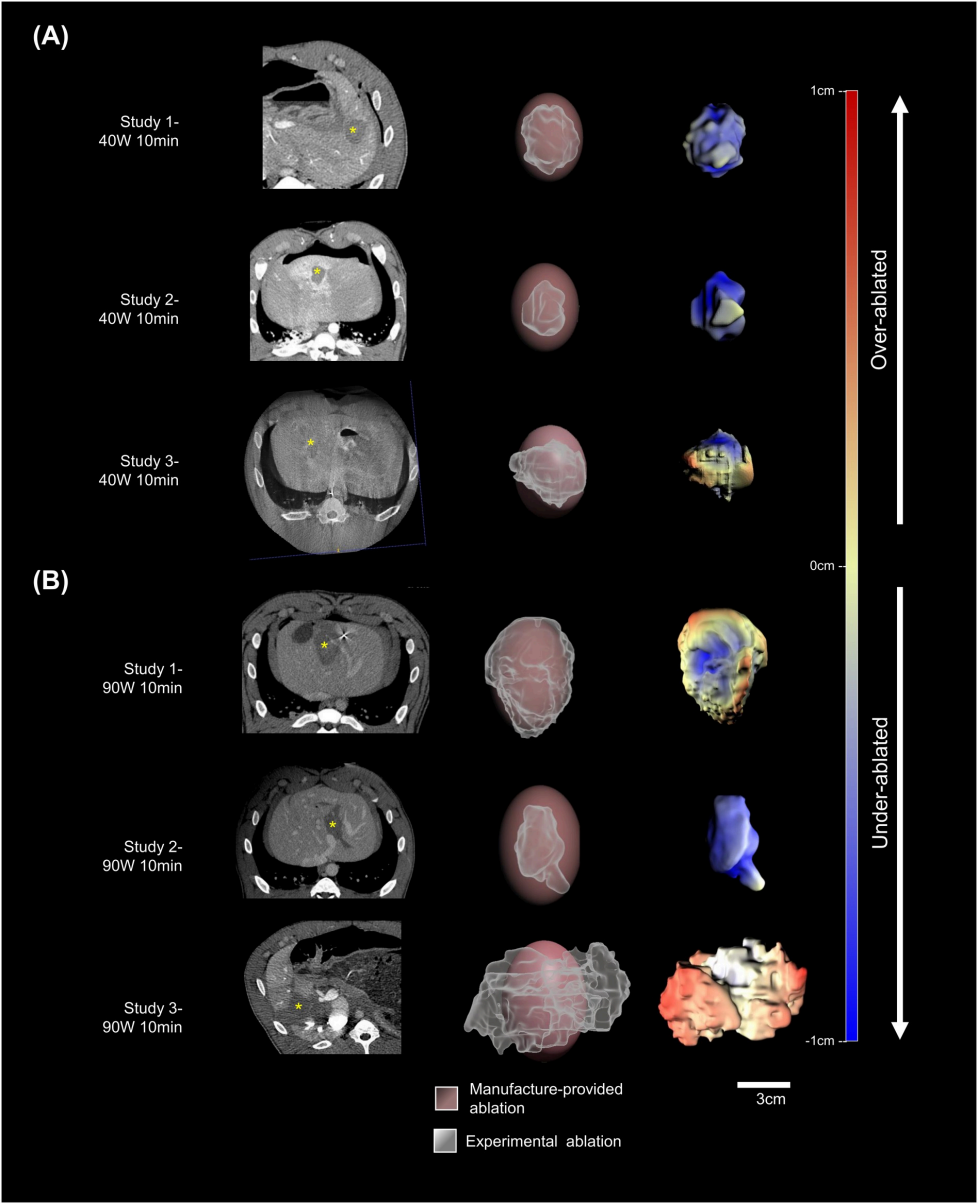
Characterization of microwave ablation zones *in vivo*. The results of (A) 40W and (B) 90W 10-minute microwave ablations *in vivo* are shown (n = 3 each). Portal phase contrast-enhanced CT (left column) after ablation demonstrating the ablation zone (*). Registered overlay of surface renderings of the manufacturer-provided (pink) and experimental segmented (white) ablation zones (middle column). The quantified differences in distance between expected and actual ablation zone surfaces are represented in the color-coded scale on the surface rendering of the ablation zone (right column). “Under-ablated” refers to a surface of the ablation zone that was inside the expected ablation zone while “over-ablated” refers to the surface of the ablation zone lying outside of the expected ablation.

The 3D segmented swine liver ablation zones co-registered with the manufacturer-provided ablation zones, revealed discrepancies between the two. The congruence of the boundaries of the *in vivo* ablation and manufacturer-provided zones varied, illustrating both “under-ablated” or “over-ablated” regions, compared to manufacturer-provided ablation zones (Fig 5). The average volume of the 40W ablations was 40% (9.8±4.8cm^3^) of the volume calculated from manufacturer-provided dimensions of 24.5cm^3^. The average volume of the 90W ablations was 91% (54.3±41.7cm^3^) of the manufacturer-provided volume of 60cm^3^ (Fig 2C, Table 1). However, in one 90W ablation, the non-perfused region was enlarged due to vascular occlusion. Excluding this value, the remaining two were 55% (33.2±28.4cm^3^) of the manufacturer-provided volume.

#### 2D and 3D ablation zone morphometrics

*In vivo* gross pathologic analysis of 2D cross-sectional slices obtained from excised ablation zones demonstrated solidity of 0.87±0.02 and 0.97±0.03 for 40W and 90W ablations (p = 0.193), respectively (Fig 2D, Table 1). 3D solidity, calculated from segmented ablation zones, showed 40W ablation solidity (0.87±0.02) was not different from 90W ablation solidity (0.75±0.15, p = 0.311).

#### Gross pathology

The heat-affected zones were consistently larger in the 90W ablations compared to the 40W ablations (Fig 2B, Table 3). The maximum area of the central necrosis zone was 1.2±0.4cm^2^ and 1.9±0.7cm^2^ for 40W and 90W ablations, respectively. The irreversible damage zone area was 1.9±0.5cm^2^ and 2.8±0.6cm^2^ for 40W and 90W ablations, respectively. The hemorrhagic halo zone area was 3.1±1.2cm^2^ and 9.1±3.0cm^2^ for 40W and 90W ablations, respectively, which is smaller than the manufacturer-provided cross-sectional area of 8.6cm^2^ and 14.5cm^2^ for the 40W and 90W ablations.

**Table 3:** Heat affected zones of 40W and 90W 10-minute ablations derived from post-mortem gross pathology.

Heat affected zone	Ablation protocol	Area (cm^2^) *Average ± SD*
**(i) central necrosis**	40W	1.17 *± 0*.*44*
	90W	1.88 *± 0*.*68*
**(ii) irreversible damage**	40W	1.86 *± 0*.*45*
	90W	2.75 *± 0*.*62*
**(iii) hemorrhagic halo**	40W	2.11 *± 0*.*45*
	90W	3.33 *± 0*.*47*

SD = standard deviation

### Preclinical case example

**Fig 6:**
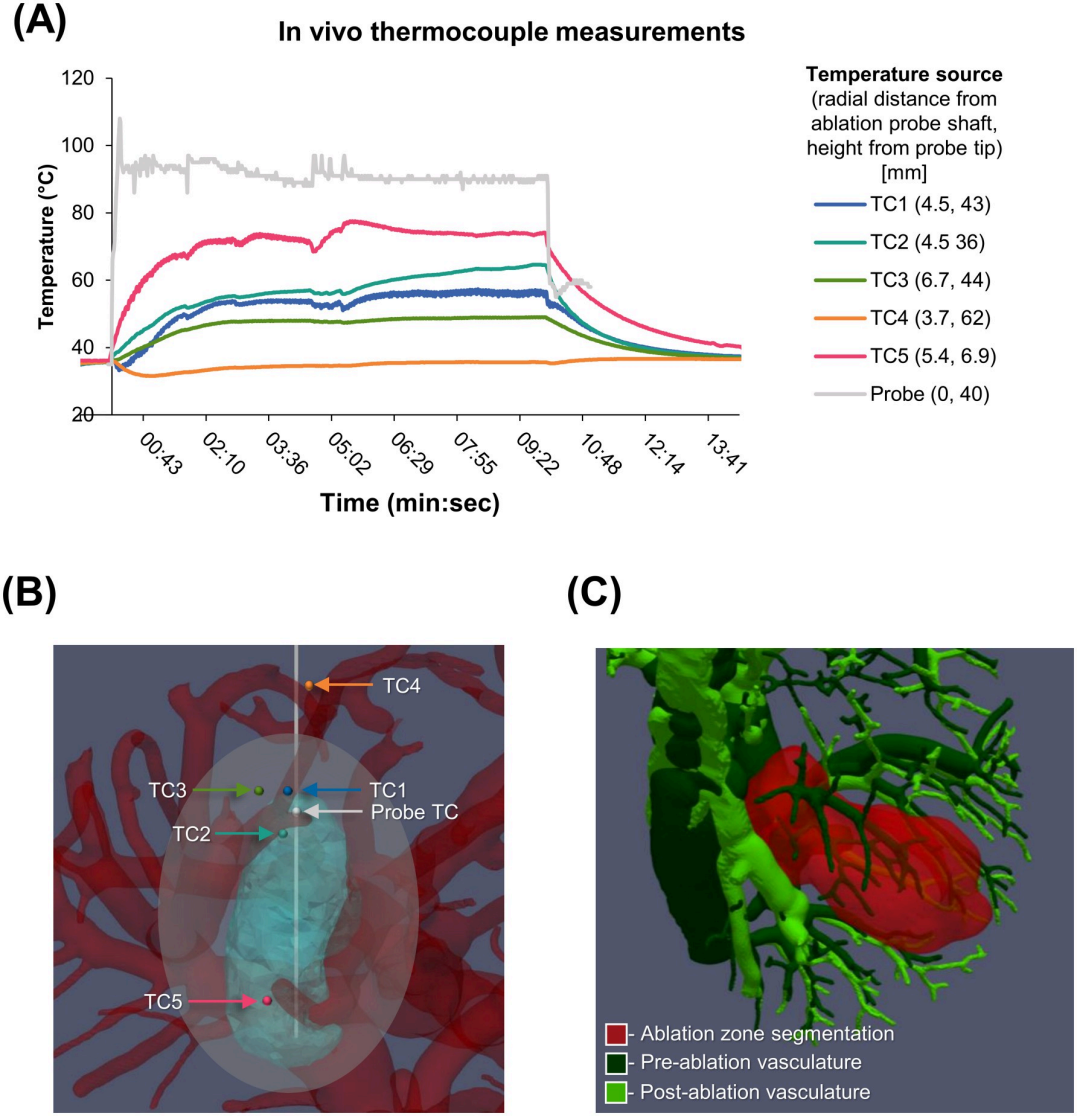
Preclinical case showing collected data and use. (A) Thermocouple measurements during a 90W 10-minute ablation with radial distance from the ablation probe and depth noted. (B) Segmented post-ablation vasculature (red) with the post-ablation un-enhancing liver (teal) co-registered with manufacturer-provided ablation zone (white). The thermocouple positions are indicated by colored dots and labeled by arrows. (C) Co-registered pre-ablation vasculature (dark green), post-ablation vasculature (light green), and the post-ablation non-enhancing volume (red) were segmented from pre- and post-ablation contrast enhanced CTs. There is loss of vascular enhancement in the region on the post-ablation CT. The latter represents the volume of thermal ablation and a distal area with significant vascular stasis. TC = thermocouple.

An informative preclinical case example of 90W ablation performed in a 59kg swine with five thermocouples placed is shown (Fig 6A). Pre- and post-ablation contrast-enhanced CT defined vasculature surrounding the ablation zone and non-enhancing tissue following ablation (Fig 6B and 6C). Thermocouples, probes, ablation zone and vasculature identified and co-registered, and temperature measurements could be correlated with isotherms modeled with treatment planning software. A volume of nonenhancing liver distal to the expected ablation is present in the distribution of vessels that also do not enhance following ablation.

## Discussion

In this study, we developed a comprehensive method for characterization of MWA and provide a detailed workflow and data to inform development of biophysical models that can better predict the dynamics of the ablation, given the device, power, probe location, and local anatomy. Both bench and *in vivo* models demonstrated differences from expected ablation zones and inter-ablation variability. These data may advance understanding of the relationships between the generation of ablative energy and organ physiology, including tissue properties, vascular flow, and perfusion. The example case illustrates the value of mapping vasculature before and after ablation to aid in interpretation of imaging findings.

Despite the widespread use of ablation, there are still significant challenges that hamper broader clinical adoption. Among these challenges, a lack of standardization and appropriate tools to evaluate the treatment results hinders the collection and comparability of reproducible standardized evidence of the treatment effectiveness. To better plan, predict, and evaluate ablation, computer-assisted ablation software is being developed [15, 29, 30]. However, current approaches include ablation zone predictions without considerations such as the heat sink effect, tissue type, nearby anatomy, system cost, a lack of integrated procedural imaging systems, dependence on a single device, or the lack of long-term, comparative, or randomized multi-center studies that demonstrate the effectiveness of tools. Ablation might be optimized or informed by thermal dose engine tools integrating biophysical model prediction of the temperature field around a thermal ablation. To improve thermal dose engine prediction, one goal of this study was to generate comprehensive *in vivo* datasets than might be used to evaluate patient-specific ablation software.

One challenge in predicting ablation zones is the definition of the ablation boundary. For *in vivo* studies, the pathologic edge of the ablation zone was defined as the outermost section or the hemorrhagic halo [21]. The ablation zone boundary implies a border where irreversible structural changes in cells causing tumor death [31]. On CT, the ablation area has been defined as low attenuation, where microvascular bleeding and coagulation can be differentiated from the surrounding perfused tissue [32]. While we reported this area as the ablation zone, Cha et al. suggested that contrast-enhanced CT overestimates the ablation due to restricted blood flow in areas adjacent to the lesion. Additionally, how this visualized area is related to critically high temperatures requires further investigation and could benefit from correlation of imaging to histopathology analysis. Studies have suggested that this critical temperature and time for coagulation and cell death in tumors is between 50 and 60°C for less than 3 minutes [33] or at a temperature range anywhere from 30 to 100°C and highly dependent on tissue type and temperature duration [34]. Although results from this study suggest that the ablation zone of the TMTCP phantom may be significantly smaller, borders visually defined in the ablated liver tissue models may not correspond as specifically as in a TMTCP phantom model at the 50–60°C range. Our study has demonstrated that points central to the ablation zone reach critical temperatures within 1 minute for both high and low power settings. How this propagates outward into an ablation zone and visually correlating ablation zone borders in both the excised swine liver and *ex vivo* bovine liver to temperature and duration requires further study.

Tissue contraction is another potential consideration when predicting and measuring MWA zone size. Water vaporization at appropriate temperatures dehydrates and contracts tissue closest to the probe, and changes in the dielectric tissue properties impact thermal conductivity [35]. Studies found that tissue contraction may be more dramatic under MWA compared to radiofrequency ablation with ablated volume contractions ranging between 10 and 38% [35, 36]. Significant tissue contraction and shrinkage may partially explain differences in expected and measured ablation zone sizes in this study. Of note, the *in vivo* ablation zones presented here are segmented from a contrast-CT taken approximately 1-hour after the ablation. Exactly how these non-enhancing volumes compare to ablation zones days to weeks post-ablation is poorly understood, but 1-hour may not fully represent the complete subsequent volume, as the ablation evolves in the initial hours to days.

In this study, morphological analyses showed higher values of solidity in bench studies compared to *in vivo* studies as the bench studies lack perfusion. Further, low power *in vivo* ablations had higher solidity and less shape variability. The trend toward lower solidity and more inconsistent shape in high power *in vivo* ablations may reflect the heat sink effect from convective heat loss, as there is increased likelihood of interaction with nearby vessels in a larger ablation volume. However, due to the low sample size in this study and the intentional placement of the ablation probe by the operator to avoid major blood vessels, a clear correlation to vessels in and adjacent to the ablation zone and the size of the ablation zone cannot be determined. In addition, the narrow liver shape in swine may predispose to heterogeneity of an infarction effect, whereby differing ablation zones are more prone to loss of dual blood supply than might occur in a human liver.

There were limitations to the study. Intraprocedural localization of thermocouples and probes was challenging and tissue contraction could have also impacted the perceived thermocouple position. Breath-holds were performed for each image, however, fiducial marker measurements from this study suggested that positions may vary up to 5mm between scans. This can be more pronounced near the diaphragm. Some thermocouples may also have been displaced during removal of the probe. Other study limitations include the small sample size in a healthy swine model and that tests were limited to two constant power settings (low and moderate) with one type of device. MWA products can differ in terms of energy delivery mode (continuous versus pulsed) and in measurements and reporting of delivered energy. This investigation was also acute, terminating 2.5 hours post-ablation. Full morphological changes in the ablated tissue and the accompanying immune response may take longer to develop. The acute examination of the tissue may underestimate the effects of ablation and examination at later time points would help to differentiate these observed changes from full morphological changes. Also, ablation size in healthy swine tissue cannot readily be extrapolated to neoplastic tissue with or without diseased liver. Moreover, swine contain multiple, thin liver lobes, which impacts the overall heat distribution and should be considered in translatin these results. Longer-term studies with a variety of power settings or devices may address some of these limitations.

Characterizing MWA towards validation of biophysical models is challenging and multifaceted. The temporal and spatial data of this study should support development of predictive ablation models to optimize treatment planning. However, applications of complex ablation models to the prediction of treatment zones and translation to improved clinical outcomes needs further study in a larger number of subjects. Clearly, with MWA variabilities, what the operator expected may not be what actually occurs or is observed on CT. However, a broader understanding of the variabilities and complex influences upon MWA treatment volumes may be requisite to clinically impactful models.

## Supporting information

S1 Table Raw data used to calculate morphometrics and display temperature curves for *ex vivo* and *in vivo* studies. (XLSX)

## References

[1] Glassberg MB, Ghosh S, Clymer JW, Wright GWJ, Ferko N, Amaral JF. Microwave ablation compared with hepatic resection for the treatment of hepatocellular carcinoma and liver metastases: a systematic review and meta-analysis. World journal of surgical oncology. 2019;17(1):98. Epub 20190610. doi: 10.1186/s12957-019-1632-6 . 31182102 PMC6558848

[2] Xu Q, Kobayashi S, Ye X, Meng X. Comparison of Hepatic Resection and Radiofrequency Ablation for Small Hepatocellular Carcinoma: A Meta-Analysis of 16,103 Patients. Scientific reports. 2014;4(1):7252. doi: 10.1038/srep07252 25429732 PMC4246212

[3] Ahmed M, Moussa M, Goldberg SN. Synergy in cancer treatment between liposomal chemotherapeutics and thermal ablation. 2012;165:424–37. doi: 10.1016/j.chemphyslip.2011.12.002 . 22197685 PMC4001764

[4] Belghiti J, Carr BI, Greig PD, Lencioni R, Poon RT. Treatment before liver transplantation for HCC. 2008;15:993–1000. doi: 10.1245/s10434-007-9787-8 . 18236111

[5] de Lope CR, Tremosini S, Forner A, Reig M, Bruix J. Management of HCC. 2012;56 Suppl 1:S75–87. doi: 10.1016/S0168-8278(12)60009-9 . 22300468

[6] Brace CL. Radiofrequency and microwave ablation of the liver, lung, kidney, and bone: what are the differences? 2009;38:135–43. doi: 10.1067/j.cpradiol.2007.10.001 . 19298912 PMC2941203

[7] Brace CL. Microwave tissue ablation: biophysics, technology, and applications. Crit Rev Biomed Eng. 2010;38(1):65–78. doi: 10.1615/critrevbiomedeng.v38.i1.60 . 21175404 PMC3058696

[8] Seitel A, Engel M, Sommer CM, Radeleff BA, Essert-Villard C, Baegert C, et al. Computer-assisted trajectory planning for percutaneous needle insertions. Med Phys. 2011;38(6):3246–59. doi: 10.1118/1.3590374 . 21815399

[9] Li K, Su Z, Xu E, Guan P, Li LJ, Zheng R. Computer-assisted hepatocellular carcinoma ablation planning based on 3-D ultrasound imaging. Ultrasound Med Biol. 2016;42(8):1951–7. Epub 20160425. doi: 10.1016/j.ultrasmedbio.2016.03.013 . 27126243

[10] Ren H, Campos-Nanez E, Yaniv Z, Banovac F, Abeledo H, Hata N, et al. Treatment planning and image guidance for radiofrequency ablation of large tumors. IEEE J Biomed Health Inform. 2014;18(3):920–8. Epub 20131024. doi: 10.1109/JBHI.2013.2287202 . 24235279 PMC4113118

[11] Ahmed M, Solbiati L, Brace CL, Breen DJ, Callstrom MR, Charboneau JW, et al. Image-guided tumor ablation: standardization of terminology and reporting criteria—a 10-year update. Radiology. 2014;273(1):241–60. Epub 20140613. doi: 10.1148/radiol.14132958 . 24927329 PMC4263618

[12] Chiang J, Loecher M, Moulin K, Meloni MF, Raman SS, McWilliams JP, et al. 4D flow MR imaging to improve microwave ablation prediction models: a feasibility study in an in vivo porcine liver. Journal of vascular and interventional radiology: JVIR. 2020;31(10):1691–6.e1. Epub 20200313. doi: 10.1016/j.jvir.2019.11.034 . 32178944 PMC7486998

[13] Chiang J, Cristescu M, Lee MH, Moreland A, Hinshaw JL, Lee FT, et al. Effects of microwave ablation on arterial and venous vasculature after treatment of hepatocellular carcinoma. Radiology. 2016;281(2):617–24. Epub 20160603. doi: 10.1148/radiol.2016152508 . 27257951 PMC5084967

[14] Bhardwaj N, Dormer J, Ahmad F, Strickland AD, Gravante G, West K, et al. Microwave ablation of the liver: a description of lesion evolution over time and an investigation of the heat sink effect. Pathology. 2011;43(7):725–31. doi: 10.1097/PAT.0b013e32834c356c . 22027742

[15] Lyons GR, Pua BB. Ablation planning software for optimizing treatment: challenges, techniques, and applications. Tech Vasc Interv Radiol. 2019;22(1):21–5. Epub 20181102. doi: 10.1053/j.tvir.2018.10.005 . 30765071

[16] Kim C. Understanding the nuances of microwave ablation for more accurate post-treatment assessment. Future Oncol. 2018;14(17):1755–64. Epub 20180214. doi: 10.2217/fon-2017-0736 . 29441813

[17] Saccomandi P, Schena E, Massaroni C, Fong Y, Grasso RF, Giurazza F, et al. Temperature monitoring during microwave ablation in ex vivo porcine livers. Eur J Surg Oncol. 2015;41(12):1699–705. Epub 20150925. doi: 10.1016/j.ejso.2015.08.171 . 26433708 PMC5513178

[18] Jelbuldina M, Korobeinyk A, Korganbayev S, Tosi D, Dukenbayev K, Inglezakis VJ. Real-time temperature monitoring in liver during magnetite nanoparticle-enhanced microwave ablation with fiber bragg grating sensors: ex vivo analysis. IEEE Sensors Journal. 2018;18(19):8005–11. doi: 10.1109/JSEN.2018.2865100

[19] Chen MH, Yang W, Yan K, Dai Y, Wu W, Fan ZH, et al. The role of contrast-enhanced ultrasound in planning treatment protocols for hepatocellular carcinoma before radiofrequency ablation. Clin Radiol. 2007;62(8):752–60. Epub 20070604. doi: 10.1016/j.crad.2006.12.013 . 17604763

[20] Hines-Peralta AU, Pirani N, Clegg P, Cronin N, Ryan TP, Liu Z, et al. Microwave ablation: results with a 2.45-GHz applicator in ex vivo bovine and in vivo porcine liver. Radiology. 2006;239(1):94–102. Epub 20060216. doi: 10.1148/radiol.2383050262 . 16484351

[21] Meloni MF, Andreano A, Bovo G, Chiarpotto B, Amabile C, Gelsomino S, et al. Acute portal venous injury after microwave ablation in an in vivo porcine model: a rare possible complication. Journal of vascular and interventional radiology: JVIR. 2011;22(7):947–51. Epub 20110508. doi: 10.1016/j.jvir.2011.03.012 . 21550820

[22] Chiang J, Hynes K, Brace CL. Flow-dependent vascular heat transfer during microwave thermal ablation. Annu Int Conf IEEE Eng Med Biol Soc. 2012;2012:5582–5. doi: 10.1109/EMBC.2012.6347259 . 23367194 PMC3563104

[23] Chiang J, Birla S, Bedoya M, Jones D, Subbiah J, Brace CL. Modeling and validation of microwave ablations with internal vaporization. IEEE Trans Biomed Eng. 2015;62(2):657–63. Epub 20141015. doi: 10.1109/TBME.2014.2363173 . 25330481 PMC4303487

[24] Liu D, Brace CL. Numerical simulation of microwave ablation incorporating tissue contraction based on thermal dose. Phys Med Biol. 2017;62(6):2070–86. Epub 20170202. doi: 10.1088/1361-6560/aa5de4 . 28151729 PMC5488337

[25] Harari CM, Magagna M, Bedoya M, Lee FT Jr., Lubner MG, Hinshaw JL, et al. Microwave ablation: comparison of simultaneous and sequential activation of multiple antennas in liver model systems. Radiology. 2016;278(1):95–103. Epub 20150702. doi: 10.1148/radiol.2015142151 . 26133361 PMC4699493

[26] Eranki A, Mikhail AS, Negussie AH, Katti PS, Wood BJ, Partanen A. Tissue-mimicking thermochromic phantom for characterization of HIFU devices and applications. Int J Hyperthermia. 2019;36(1):518–29. doi: 10.1080/02656736.2019.1605458 . 31046513 PMC6625350

[27] Mikhail AS, Negussie AH, Graham C, Mathew M, Wood BJ, Partanen A. Evaluation of a tissue-mimicking thermochromic phantom for radiofrequency ablation. Med Phys. 2016;43(7):4304. doi: 10.1118/1.4953394 . 27370145 PMC4912564

[28] Boyd S, Boyd SP, Vandenberghe L. Convex optimization: Cambridge university press; 2004.

[29] Vo Chieu VD, Wacker F, Rieder C, Pöhler GH, Schumann C, Ballhausen H, et al. Ablation zone geometry after CT-guided hepatic microwave ablation: evaluation of a semi-automatic software and comparison of two different ablation systems. International Journal of Hyperthermia. 2020;37(1):533–41. doi: 10.1080/02656736.2020.1766704 32468872

[30] Beermann M, Lindeberg J, Engstrand J, Galmén K, Karlgren S, Stillström D, et al. 1000 consecutive ablation sessions in the era of computer assisted image guidance—Lessons learned. Eur J Radiol Open. 2019;6:1–8. Epub 20181205. doi: 10.1016/j.ejro.2018.11.002 . 30547062 PMC6282637

[31] Sapareto SA, Dewey WC. Thermal dose determination in cancer therapy. Int J Radiat Oncol Biol Phys. 1984;10(6):787–800. doi: 10.1016/0360-3016(84)90379-1 . 6547421

[32] Cha CH, Lee FT Jr., Gurney JM, Markhardt BK, Warner TF, Kelcz F, et al. CT versus sonography for monitoring radiofrequency ablation in a porcine liver. AJR Am J Roentgenol. 2000;175(3):705–11. doi: 10.2214/ajr.175.3.1750705 . 10954454

[33] Heisterkamp J, van Hillegersberg R, IJ JN. Critical temperature and heating time for coagulation damage: implications for interstitial laser coagulation (ILC) of tumors. Lasers Surg Med. 1999;25(3):257–62. doi: 10.1002/(sici)1096-9101(1999)25:3&lt;257::aid-lsm10&gt;3.0.co;2-v . 10495303

[34] Ahmed M, Brace CL, Lee FT Jr., Goldberg SN. Principles of and advances in percutaneous ablation. Radiology. 2011;258(2):351–69. doi: 10.1148/radiol.10081634 . 21273519 PMC6939957

[35] Brace CL, Diaz TA, Hinshaw JL, Lee FT Jr. Tissue contraction caused by radiofrequency and microwave ablation: a laboratory study in liver and lung. Journal of vascular and interventional radiology: JVIR. 2010;21(8):1280–6. Epub 20100527. doi: 10.1016/j.jvir.2010.02.038 20537559 PMC2920145

[36] Erxleben C, Niehues SM, Geyer B, Poch F, Bressem KK, Lehmann KS, et al. CT-based quantification of short-term tissue shrinkage following hepatic microwave ablation in an in vivo porcine liver model. Acta Radiol. 2021;62(1):12–8. Epub 20200407. doi: 10.1177/0284185120914452 32264686

